# Homozygous *FANCM* Variant c.5101C>T p.(Gln1701*) in a Patient With Early Onset Breast Cancer, Chemotherapy Toxicity, and Chromosome Fragility: A Case Report

**DOI:** 10.1002/cnr2.70283

**Published:** 2025-08-20

**Authors:** Sonja Sulkava, Anna H. Hakonen, Rikke Christensen, Minna Pöyhönen, Heli Nevanlinna

**Affiliations:** ^1^ Department of Clinical Genetics, HUS Diagnostic Center University of Helsinki and Helsinki University Hospital Helsinki Finland; ^2^ Department of Public Health Finnish Institute for Health and Welfare Helsinki Finland; ^3^ Department of Clinical Genetics Aarhus University Hospital Aarhus Denmark; ^4^ European Reference Network on Genetic Tumour Risk Syndromes (ERN GENTURIS) Helsinki Finland; ^5^ Laboratory of Genetics, HUS Diagnostic Center University of Helsinki and Helsinki University Hospital Helsinki Finland; ^6^ Department of Obstetrics and Gynecology University of Helsinki and Helsinki University Hospital Helsinki Finland

**Keywords:** breast cancer, c.5101C>T p.(Gln1701*), chemotherapy toxicity, chromosome fragility, FANCM gene, gonadal dysfunction

## Abstract

**Background:**

Biallelic *FANCM* variants are linked to a Fanconi anemia‐like cancer predisposition syndrome, which includes early onset breast cancer, chemotherapy toxicity, and chromosome fragility. Additionally, heterozygous truncating variants have been linked to increased breast cancer risk. However, the published results have been inconsistent, and the risks and the functional effects associated with the variants also vary depending on the position in the gene, with N‐terminal truncating variants having a stronger effect. Compared to other *FANCM* variants studied, milder patient phenotypes and only late onset breast cancer have been reported for the homozygous C‐terminal c.5101C>T variant, which is enriched in Finland.

**Case:**

We report here a Finnish patient, homozygous for the *FANCM* c.5101C>T p.(Gln1701*) variant, who manifested with early onset triple‐negative breast cancer, chemotherapy toxicity, and chromosome fragility. Homozygosity for c.5101C>T has previously been reported in two Finnish siblings with primary ovarian insufficiency and chromosome fragility.

**Conclusion:**

These findings suggest that the C‐terminal *FANCM* variant c. 5101C>T may also be linked to a phenotype similar to the phenotype associated with N‐terminal truncating variants when inherited in a homozygous state.

## 1 Introduction

The *FANCM* gene has been suggested as a predisposition gene for breast cancer [1, 2, 3]. However, the results on heterozygous truncating variants from different case‐control studies have been inconsistent [4, 5]. The C‐terminal NM_020937.2:c.5101C>T p.(Gln1701*) variant, located in exon 20/23, is enriched in Finland with a carrier frequency of almost 2% (MAF 0.009339), but rare in other populations (gnomAD v4.0.0, [6]). We previously found the variant associated with a moderate risk for breast cancer, especially for triple‐negative and familial breast cancer [1]. However, the extensive Breast Cancer Association Consortium (BCAC) study only found the N‐terminal variant c.1972C>T p.(Arg658*) in exon 11 significantly associated with ER‐negative and triple‐negative breast cancer, with no evidence for c.5101C>T and only weak evidence for another C‐terminal variant c.5791C>T p.(Arg1931*) for ER‐negative breast cancer [4].

In vitro functional studies have shown that all three variants were deleterious to their capacity to rescue cell survival in *FANCM* knockdown fibroblasts exposed to the DNA‐damaging agent diepoxybutane (DEB). However, the effect of the c.1972C>T was more detrimental than that of the two C‐terminal variants, which had intermediate effects only. The c.1972C>T cell line also showed a higher number of chromatid breaks than the other two variants [4]. At the RNA level, all of these variants lead to stable messenger RNA (mRNA); furthermore, the *FANCM* alleles exhibiting the c.5101C>T and c.5791C>T variants have been shown to produce truncated proteins [4].

FANCM protein is a component of the Fanconi anemia (FA) core complex, but *FANCM* is not a canonical FA gene. FA is a hereditary syndrome which, in the classical form, includes susceptibility to several cancers, chemotherapy toxicity, bone marrow failure, congenital malformations, and chromosome fragility. However, biallelic *FANCM* variants are suggested to cause a FA‐like cancer predisposition syndrome characterized by early onset breast cancer, chemotherapy toxicity, and chromosome fragility [7, 8, 9, 10]. The breast cancer risk and functional effects of the truncating variants are considered to depend on their location within the *FANCM* gene, with N‐terminal loss of function (LoF) variants associated with a more detrimental effect than the C‐terminal LoF variants. However, population‐specific genetic risk modifiers have also been suggested [11].

Patients homozygous for the c.1972C>T variant have been reported with early onset breast cancer, whereas those homozygous for variants in the C‐terminal have had late onset breast cancer [8, 12]. Chemotherapy toxicity has been common to all genotypes [8]. Cancer patients with biallelic *FANCM* variants have also suffered from premature menopause or diminished ovarian reserve [8, 10] with familial primary ovarian insufficiency reported for Finnish carriers of the homozygous c.5101C>T variant (POI, MIM #6180969) [13].

Sensitivity to DNA interstrand crosslinking agents (such as mitomycin C and DEB) using the chromosome fragility test was demonstrated in cell lines of the patients homozygous for N‐terminal variants, such as c.1972C>T, but not in cell lines of c.5791C>T homozygotes [7, 8]. The Finnish sisters, but not a Swedish patient homozygous for c.5101C>T, showed chromosome fragility [8, 13].

We report here a Finnish breast cancer patient homozygous for *FANCM* c.5101C>T p.(Gln1701*) diagnosed with triple‐negative breast cancer at the age of 35 years. This represents the first published case of early onset breast cancer in a patient homozygous for the variant, who additionally exhibited chemotherapy toxicity and confirmed chromosome fragility.

## 2 Case Presentation

The patient is a 43‐year‐old Finnish woman who reported no previous long‐term illnesses or regular use of medication. She is an ex‐smoker with a 17‐year smoking history. The patient's family history included a mother who was diagnosed with hormone receptor‐positive breast cancer at an older age. Both her father and brother appeared to be in good health. No parental consanguinity was reported.

At the age of 35 years, the patient was diagnosed with ductal Grade III triple‐negative breast cancer (T2N0M0, MIB1 90). Treatment included mastectomy (August 2016) and chemotherapy. Later she underwent preventive mastectomy of the contralateral breast. Chemotherapy entailed docetaxel and capecitabine three times followed by CEX (cyclophosphamide, epirubicin, and capecitabine) three times. In the first cycle with docetaxel and capecitabine, the patient developed neutropenia, fever, diarrhea, and stomach cramping. She was hospitalized and treated with antibiotics and leukocyte growth factor. For the second cycle, the capecitabine dose was reduced and administered together with the leukocyte growth factor, which was tolerated. After the first cycle of CEX, the patient developed a neutropenic infection with severe neutropenia and was hospitalized. The treatment was continued as CEF (cyclophosphamide, epirubicin, and fluorouracil) together with leukocyte growth factor, but the patient developed sepsis and the treatment was interrupted. Finally, the patient was treated with two AC (doxorubicin and cyclophosphamide) dose‐dense cycles, which she tolerated well. She had no previous history of sensitivity to infections.

Before the commencement of treatment, the patient underwent a gynecologic evaluation after reporting a long history of oral contraceptive use. The ultrasonography study showed small dense ovaries with few follicles. Later, at the age of 37 years, she was diagnosed with oligomenorrhea and at the age of 40 years, FSH was in the post‐menopausal level. A diagnosis of premature ovarian failure could not be confirmed because of the confounding effect of chemotherapy on fertility. The clinical status showed no structural abnormalities or a short stature suggestive of FA. Development has been normal. Thorough hematological examinations (including a bone marrow sample) did not reveal abnormalities.

In August 2017, a multigene hereditary breast and ovarian cancer panel identified a homozygous *FANCM* variant c.5101C>T p.(Gln1701*) (NM_020937.2), which was reported as a variant of uncertain significance (VUS). DNA was extracted from peripheral blood and screened using a next‐generation sequencing‐based (NGS) Ion Torrent semiconductor technology (28 genes, Table S1) at the Laboratory of Genetics in the Helsinki University Hospital, a FINAS (Finnish Accreditation Service) accredited facility. Of the target areas, 99.5% reached the coverage of 30×. The variant was confirmed with Sanger sequencing. Sanger sequencing also confirmed the carrier status in the patient's mother with late‐onset breast cancer.

After a new contact to the Clinical Genetics Unit of the Helsinki University Hospital, a diagnostic chromosome breakage test was performed in December 2022 at the laboratory of the Department of Clinical Genetics at Aarhus University Hospital, Denmark, which is accredited by the Danish Accreditation Fund, DANAK. Chromosome breakage analysis was performed essentially as described [14]. Briefly, peripheral lymphocytes from the patient and a healthy control were cultured in complete medium (PB‐MAX, Gibco by Life Technologies) with and without mitomycin C at a concentration of 174 nM for 72 h. Colcemid (Biowest) was added at a final concentration of 0.17 μg/mL, and after 35 min, cells were harvested and metaphase spreads were prepared using standard procedures. Chromosomes were stained using Q‐banding, and the number of chromosome breaks was counted in 20 metaphases from each sample except the healthy control without mitomycin C, where only five cells were analyzed. When more than 10 breaks were observed in a metaphase, the precise number of breaks was not determined. Instead, the metaphase was classified as having more than 10 breaks. Statistical significance was assessed with Fisher's exact test.

**FIGURE 1:**
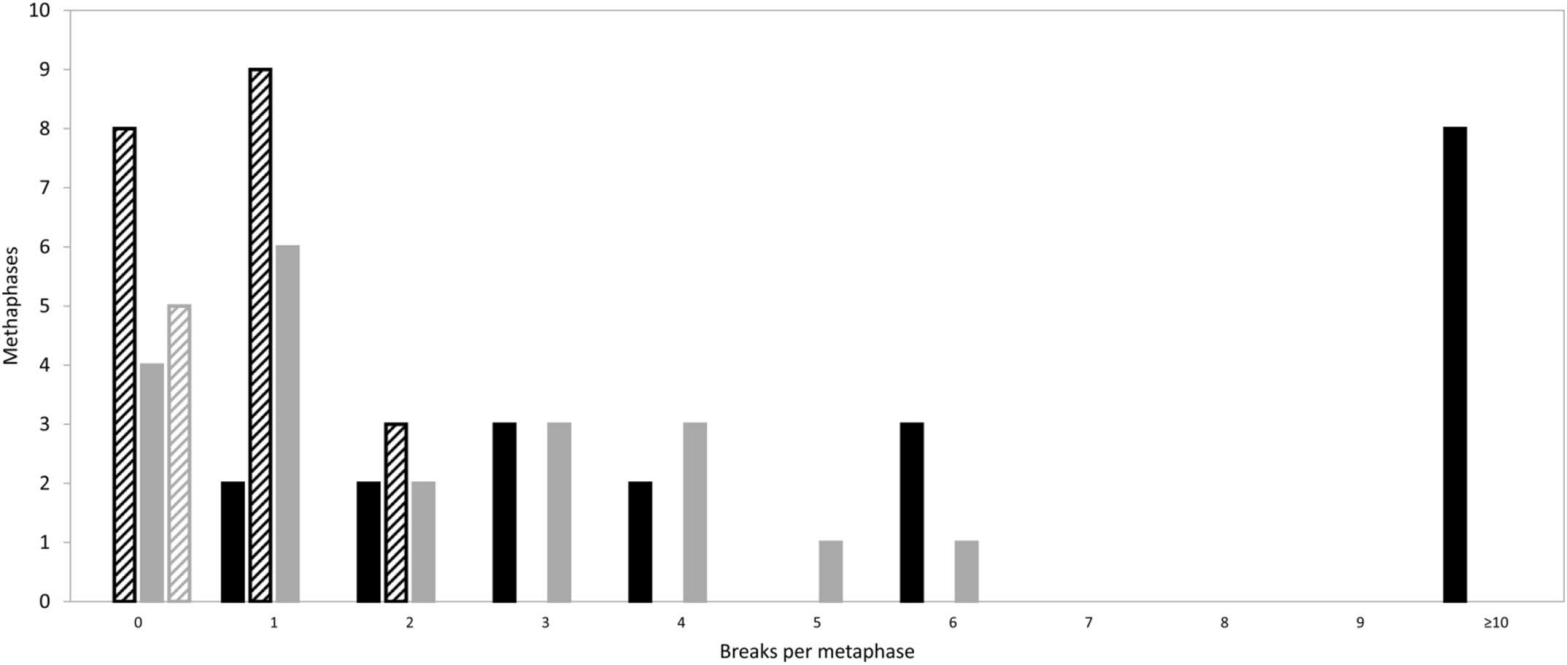
Breaks per metaphase in the chromosome breakage analysis for the patient sample and control sample. Histogram showing number of chromosome breaks per metaphase after culturing of peripheral lymphocytes from the patient (black columns) and a healthy control (grey columns) with (filled columns) and without (hatched columns) mitomycin C.

**FIGURE 2:**
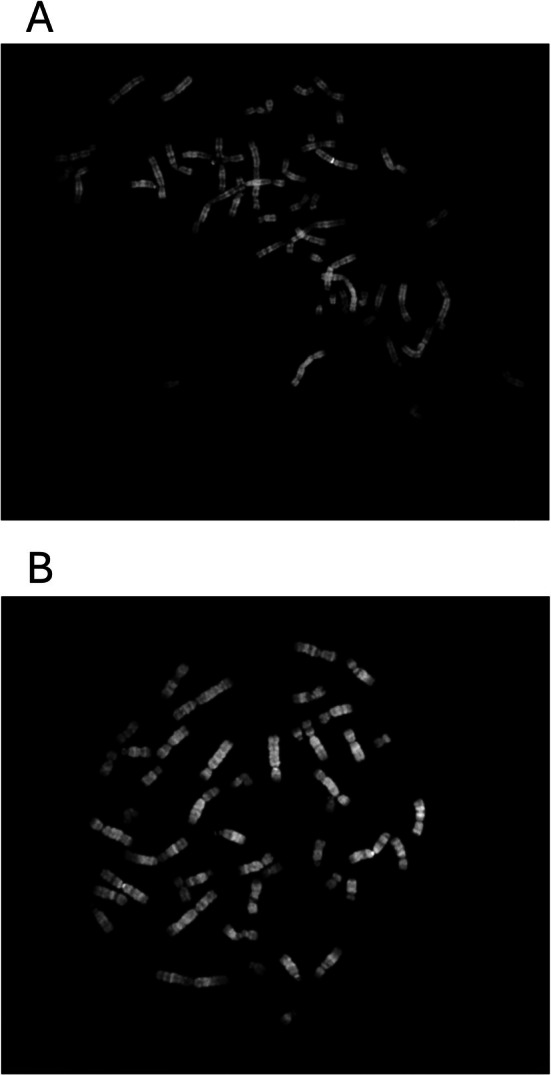
A typical metaphase from the patient sample after the induction of chromosome breakage using mitomycin C (A) and from the healthy control cultured without mitomycin C (B). Note the large numbers of chromosome breakages/fragmentation of chromosomes in A.

The results of the chromosome breakage test revealed that the patient sample without mitomycin C treatment showed 0–2 spontaneous breaks per metaphase, whereas the culture from a healthy control without mitomycin C did not show any breaks (Figure 1). Treatment of patient and control cells with mitomycin C showed that patient cells were significantly more sensitive to mitomycin C treatment than control cells (*p* = 0.003). In 8/20 metaphases from patient cells treated with mitomycin C, more than 10 breaks were detected, whereas no metaphases had more than 10 breaks in control cells treated with mitomycin C (Figure 1). Figure 2 shows typical metaphases from the patient and control samples. The results were considered abnormal and compatible with FA.

In June 2023, to study potential alternative causes for the observed chromosome fragility, a hematological gene panel of 297 genes, including all the FA genes, was performed using skin biopsy‐derived DNA (Table S2). To exclude other causative genetic factors, the analysis was further expanded to clinical diagnostic exome sequencing in the Laboratory of Genetics in the Helsinki University Hospital (Supporting Information Methods). The analysis also included NGS‐based analysis for copy number variations (CNV's). No pathogenic, likely pathogenic, or VUS variants were reported other than the previously identified *FANCM* c.5101C>T in a homozygous state.

Clinical and laboratory data were collected from the medical records of the Helsinki University Hospital. The study was approved by the Ethical Review Board of Helsinki University Hospital (HUS/1597/2016). The patient gave her written informed consent for the publication of case details.

## 3 Discussion

Our patient with the homozygous C‐terminal *FANCM* variant c.5101C>T shows multiple phenotypes previously related to the *FANCM* gene and especially its N‐terminal biallelic truncating variants. She was diagnosed with triple‐negative breast cancer at the age of 35 years. Upon treatment, she developed severe chemotherapy toxicity, and her lymphocytes showed chromosome fragility.

The *FANCM* gene (FAAP250) was found to belong to the FA core complex and initially determined as a new FA complementation group M [15]. However, subsequent studies show that *FANCM* is not a bona fide FA gene, but its biallelic variants are linked to a milder, FA‐like cancer‐prone syndrome without congenital malformations [7, 8, 9, 10]. *FANCM* plays a role in DNA damage response and repair [16] and in functional studies, mutated cells show sensitivity to ICL agents, leading to chromosome breakage, typical also in FA.

Chromosome fragility has been consistently demonstrated in patient cell lines with biallelic truncating *FANCM* variants in the N‐terminus [7, 8]. However, the effects of C‐terminal *FANCM* variants, especially the c.5101C>T variant, have remained unclear and considered less severe due to the production of truncated FANCM with residual function [4]. Chromosome fragility has previously been linked to the biallelic c.5101C>T variant only in two Finnish sisters with POI [13]. Furthermore, lack of sensitivity to interstrand crosslinking agents in the chromosome fragility test has been reported in one Swedish patient homozygous for c.5101C>T [8]. Our findings now corroborate previous findings of chromosome fragility and underline the importance of considering this chromosome fragility phenotype in all patients homozygous for the c.5101C>T variant.

Chemotherapy toxicity has also been reported in relation to FA as well as in homozygotes of truncating *FANCM* variants, including c.5101C>T [8, 9]. In the case reported here, the patient also suffered from chemotherapy toxicity, but she benefited from reduced doses and modified agents. In heterozygous form, the c.5101C>T variant has previously been suggested to associate with worse breast cancer survival; however, the carrier patients may benefit from radiotherapy treatment more than non‐carriers [17].

A recent study has proposed the existence of potential genetic modifiers in the Finnish population, considering the breast cancer risk associated with the heterozygous *FANCM* c.5101C>T variant [11]. The presence of such modifiers could explain the earlier age of onset of the disease in our patient compared to the *FANCM* c.5101C>T homozygotes reported outside of Finland [8, 12]. Future epidemiological studies of the Finnish population however are needed to test this hypothesis.

In addition to early onset breast cancer, biallelic germline variants have been reported in several other cancers as well, especially head and neck squamous cell carcinoma, often with severe treatment‐associated chemo‐ and radiotherapy toxicity [7, 8, 12].

Previously, homozygous or biallelic *FANCM* variants have been linked to POI in several, but not all, women studied, including Finnish cases with *FANCM* c.5101C>T homozygosity ([8, 13, 18]). Our patient furthermore had a relatively early menopause in her 40s. However, we cannot rule out the possibility that it would be secondary to chemotherapy treatment and history of smoking in this case. Ultrasound study suggested dense ovaries with few follicles already before the chemotherapy, but this also may be related to the history of oral contraceptive use.

## 4 Conclusion

This case report, together with the previous report on POI in Finnish cases with the homozygous *FANCM* c.5101C>T [13], suggests that also the C‐terminal *FANCM* variant c. 5101C>T, relatively common in Finland, may be linked to early onset breast cancer, chemotherapy toxicity, and chromosome fragility as well as primary ovarian insufficiency when inherited in homozygous state.

The frequency of Finnish *FANCM* c.5101C>T homozygotes is approximately 1/10 000 which implies nearly 500 Finns living with a homozygous genotype. Our findings demonstrate the importance of diagnostic genetic testing to report the homozygous c.5101C>T variant, as the biallelic genotype may warrant cancer follow‐up or tailoring of chemotherapy treatment.

## Author Contributions

**Sonja Sulkava:** conceptualization, investigation, writing – original draft preparation, writing – review and editing. **Anna H. Hakonen:** writing – review and editing. **Rikke Christensen:** investigation, visualization, writing – review and editing. **Minna Pöyhönen:** supervision, writing – review and editing. **Heli Nevanlinna:** conceptualization, supervision, writing – original draft preparation, writing – review and editing.

## Consent

The patient gave her consent to this report.

## Conflicts of Interest

The authors declare no conflicts of interest.

## Supporting information


**Data S1.** Supporting Information.

## Data Availability

Data sharing is not applicable to this article as no new data were created or analyzed in this study.
