# Supplementary material for: Homozygous FANCM Variant c.5101C>T p.(Gln1701*) in a Patient With Early Onset Breast Cancer, Chemotherapy Toxicity, and Chromosome Fragility: A Case Report
Source: Cancer Rep (Hoboken). 2025 Aug 20;8(8):e70283. doi: 10.1002/cnr2.70283 (PMC12365610; doi:10.1002/cnr2.70283)
Supplement: Supplementary file 1 — Data S1. Supporting Information. [file CNR2-8-e70283-s001.docx]

**Supplementary material**

**Supplementary Methods**

**Exome sequencing and hematological gene panel**

Exome sequencing was performed in the Laboratory of Genetics in the Helsinki University Hospital, which is a FINAS (Finnish Accreditation Service) accredited testing laboratory.

Library preparation and enrichment was performed using a customized Twist Human Core Exome kit which included additional clinically important gene loci. The amplified libraries were sequenced with Illumina Novaseq 6000. The sequencing data were aligned to the reference human genome GRCh38. The exome sequencing showed 97.1% of regions with coverage >10x, 95.5% with coverage >30x and 87.6% with coverage >50x (the minimum criteria of 95 % of regions with coverage >30x). The median depth of sequencing was 91x. The sequencing raw data was analyzed bioinformatically. The programs used were Bcl2fastq2, trimmomatic, BWA-MEM, Genome Analysis Toolkit, CODEX and ExomeDepth. The single nucleotide variants and indels were annotated with Variant Effect Predictor (107) and CNV’s with Allissa Interpret (5.4.2). The analysis detected insertions and deletions <150 bp and intronic variants +/- 8 bp from the splicing cite junction. In addition, some previously reported deeper intronic variants were covered. Exome sequencing included CNV analysis targeted to the coding regions and was able to detect deletions and duplications of one exon or larger. Class 3 to 5 variants (interpreted using the ACMG criteria) were reported in cases where the gene identified was associated with the reported phenotype.

The hematological panel was based on the exome sequencing data. The genes included in the panel are reported in the Supplementary Table S1. Fanconi anemia genes are included in the gene panel.

**Supplementary Table S1**. Genes included in the hereditary breast and ovarian cancer gene panel performed in the Laboratory of Genetics in the Helsinki University Hospital.

| Gene |
| --- |
| BRCA1* |
| BRCA2* |
| TP53 |
| PTEN |
| STK11 |
| CDH1 |
| BLM |
| DICER1 |
| PALB2 |
| CHEK2 |
| RAD51C |
| RAD51D |
| BRIP1 |
| PPM1D |
| MLH1 |
| MSH2 |
| MHS6 |
| PMS2 |
| BARD1 |
| FANCM |
| MCPH1 |
| RAD50 |
| MRE11 |
| NBN |
| RAD51 |
| RAD51B |
| RECQL |
| XRCC2 |

*Multiplex-ligation-dependent probe amplification analysis (MLPA) of *BRCA1* and *BRCA2* was included in the gene panel.

Copy number variation of all the genes was studied as part of the exome sequencing.

**Supplementary Table S2.** Hematological gene panel in the Laboratory of Genetics in the Helsinki University Hospital.

| ABCB7 |
| --- |
| ABCG5 |
| ABCG8 |
| ACD |
| ACTN1 |
| ADA2 |
| ADAMTS13 |
| AK1 |
| AK2 |
| ALAS2 |
| AMN |
| ANK1 |
| ANKRD26 |
| ANO6 |
| AP3D1 |
| ARPC1B |
| ASXL1 |
| ATM |
| ATR |
| ATRX |
| BLM |
| BLOC1S3 |
| BLOC1S6 |
| BRAF |
| BRCA1 |
| BRCA2 |
| BRIP1 |
| CBL |
| CD46 |
| CDAN1 |
| CDC42 |
| CDIN1 |
| CDKN2A |
| CEBPA |
| CHEK2 |
| CLCN7 |
| CLPB |
| CSF2RA |
| CSF3R |
| CTC1 |
| CUBN |
| CXCR4 |
| CYB5R3 |
| CYCS |
| DDX41 |
| DHFR |
| DIAPH1 |
| DKC1 |
| DNAJC21 |
| DNASE2 |
| DNMT3A |
| DTNBP1 |
| EFL1 |
| EGLN1 |
| ELANE |
| EPAS1 |
| EPB41 |
| EPB42 |
| EPOR |
| ERCC4 |
| ERCC6L2 |
| ETV6 |
| EZH2 |
| F10 |
| F11 |
| F12 |
| F13A1 |
| F13B |
| F2 |
| F5 |
| F7 |
| F8 |
| F9 |
| FADD |
| FANCA |
| FANCB |
| FANCC |
| FANCD2 |
| FANCE |
| FANCF |
| FANCG |
| FANCI |
| FANCL |
| FANCM |
| FAS |
| FASLG |
| FERMT3 |
| FGA |
| FGB |
| FGG |
| FLI1 |
| FLNA |
| FLT3 |
| FYB1 |
| G6PC3 |
| G6PD |
| GALE |
| GATA1 |
| GATA2 |
| GBA |
| GCLC |
| GFI1 |
| GFI1B |
| GGCX |
| GINS1 |
| GP1BA |
| GP1BB |
| GP6 |
| GP9 |
| GPI |
| GSS |
| HAX1 |
| HBA1 |
| HBA2 |
| HBB |
| HFE |
| HOXA11 |
| HPS1 |
| HPS3 |
| HPS4 |
| HPS5 |
| HPS6 |
| HRAS |
| IDH1 |
| IDH2 |
| IFNGR2 |
| IKZF1 |
| IKZF2 |
| IKZF5 |
| ITGA2B |
| ITGB3 |
| ITK |
| JAGN1 |
| JAK2 |
| KDM1A |
| KDSR |
| KIF23 |
| KIT |
| KLF1 |
| KRAS |
| LAMTOR2 |
| LIG4 |
| LMAN1 |
| LPIN2 |
| LYST |
| MAD2L2 |
| MAGT1 |
| MBD4 |
| MCFD2 |
| MECOM |
| MLH1 |
| MMUT |
| MPIG6B |
| MPL |
| MPO |
| MRE11 |
| MRTFA |
| MSH2 |
| MSH6 |
| MTHFD1 |
| MTR |
| MYD88 |
| MYH9 |
| MYO5A |
| MYSM1 |
| NAF1 |
| NBEAL2 |
| NBN |
| NF1 |
| NHP2 |
| NOP10 |
| NOTCH1 |
| NPM1 |
| NRAS |
| NT5C3A |
| P2RY12 |
| PALB2 |
| PARN |
| PAX5 |
| PCCA |
| PCCB |
| PDGFRA |
| PGM3 |
| PHF6 |
| PIEZO1 |
| PKLR |
| PLA2G4A |
| PLAU |
| PMS2 |
| POT1 |
| PRF1 |
| PRKACG |
| PROC |
| PROS1 |
| PTEN |
| PTPN11 |
| PTPRJ |
| PUS1 |
| RAB27A |
| RAC2 |
| RAD50 |
| RAD51 |
| RAD51C |
| RASGRP2 |
| RBBP6 |
| RBM8A |
| RECQL4 |
| REN |
| RHAG |
| RIT1 |
| RMRP |
| RNF168 |
| RNU4ATAC |
| RPA1 |
| RPL11 |
| RPL15 |
| RPL26 |
| RPL27 |
| RPL31 |
| RPL35A |
| RPL5 |
| RPS10 |
| RPS14 |
| RPS17 |
| RPS19 |
| RPS24 |
| RPS26 |
| RPS27 |
| RPS28 |
| RPS29 |
| RPS7 |
| RTEL1 |
| RUNX1 |
| SAMD9 |
| SAMD9L |
| SBDS |
| SBF2 |
| SEC23B |
| SERPINC1 |
| SERPINF2 |
| SH2B3 |
| SH2D1A |
| SLC11A2 |
| SLC19A2 |
| SLC25A38 |
| SLC37A4 |
| SLC46A1 |
| SLC4A1 |
| SLFN14 |
| SLX4 |
| SMARCD2 |
| SOS1 |
| SPTA1 |
| SPTB |
| SRC |
| SRP54 |
| SRP72 |
| STAT3 |
| STIM1 |
| STX11 |
| STXBP2 |
| TAZ |
| TBXA2R |
| TBXAS1 |
| TCIRG1 |
| TCN2 |
| TEK |
| TERC |
| TERT |
| TET2 |
| TF |
| THBD |
| THPO |
| TINF2 |
| TMPRSS6 |
| TP53 |
| TPI1 |
| TPP1 |
| TRNT1 |
| TSR2 |
| TUBB1 |
| UBE2T |
| UNC13D |
| USB1 |
| VHL |
| VKORC1 |
| VPS13B |
| VPS45 |
| VWF |
| WAS |
| WDR1 |
| WIPF1 |
| WRAP53 |
| WT1 |
| XIAP |
| XRCC2 |
| YARS2 |
